# Supplementary material for: Maternal Immune Activation Induces Adolescent Cognitive Deficits Preceded by Developmental Perturbations in Cortical Reelin Signalling
Source: Biomolecules. 2023 Mar 7;13(3):489. doi: 10.3390/biom13030489 (PMC10046789; doi:10.3390/biom13030489)
Supplement: Supplementary file 1 [file biomolecules-13-00489-s001.zip › biomolecules-2208439-supplementary.pdf]

Appendix A: Supplementary Methods (Supplement 1)

1. Timeline of Experiment

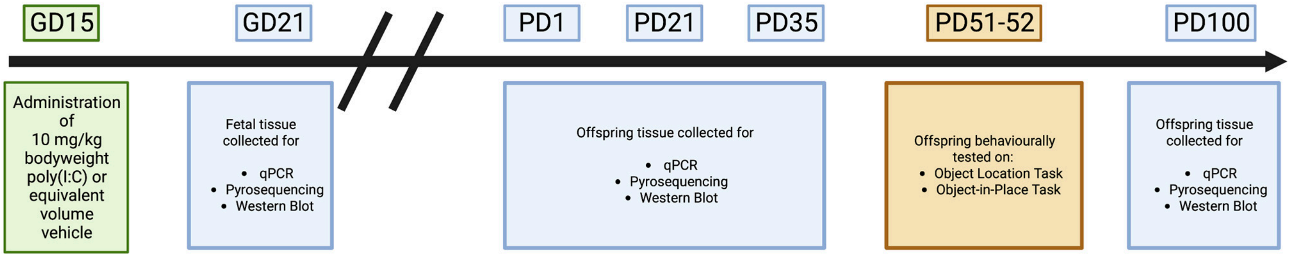

Supplementary Figure S1. Timeline of Experiment. The figure above represents the timing of procedures across the developmental timeline. Image was created with BioRender.com

2. Bisulphite-PCR (bis-PCR) agarose gel electrophoresis

2% (w/v) agarose in 1XTris-Acetate-EDTA (TAE) gels, containing 1:10,000 gel red stain. Electrophoreses performed for 2 h at 120V. Gels were imaged using the GelDoc XR imager (Bio-Rad, Watford, UK) under standard UV conditions.

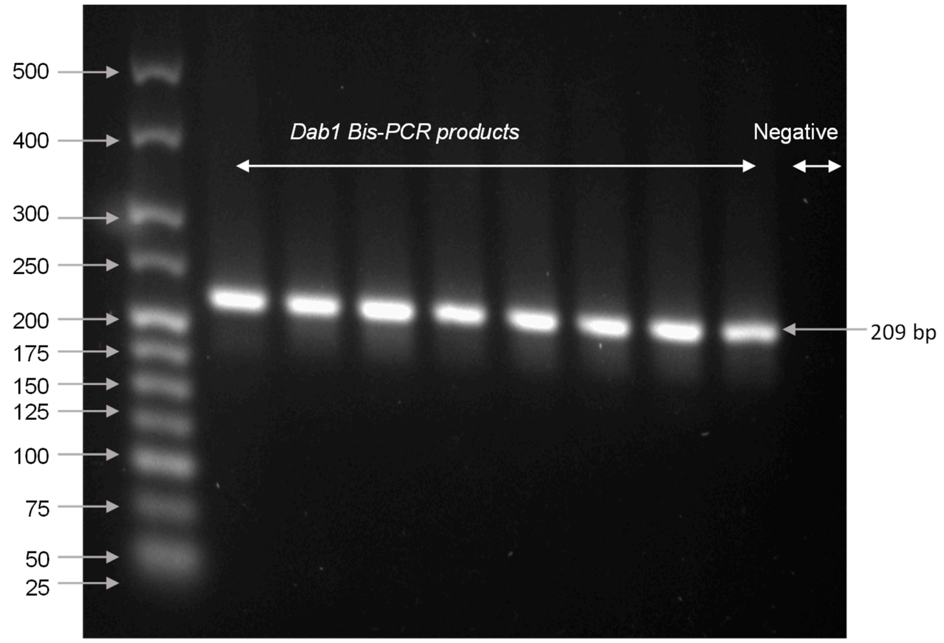

Supplementary Figure S2A. Representative agarose electrophoresis gel for *Dab1* bis-PCR products. Lane 1 contains a 25 base pair (bp) Hyperladder (Bioline, London, UK) as a molecular marker. The next eight lanes contain representative *Dab1* bis-PCR products, which amplified at the predicted amplicon size of 209bp. The final lane contains the negative control.

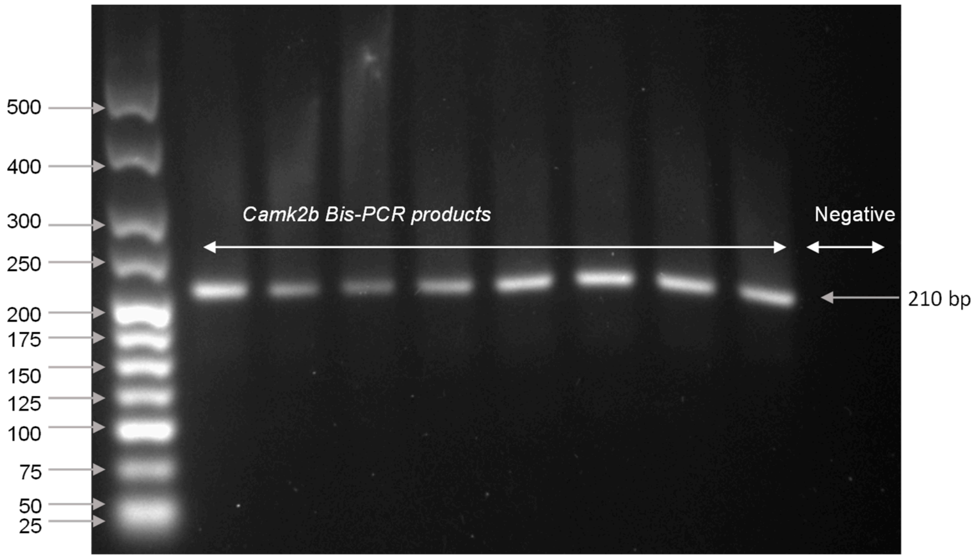

Supplementary Figure S2B. Representative agarose electrophoresis gel for *Camk2b* bis-PCR products. Lane 1 contains a 25 base pair (bp) Hyperladder (Bioline, London, UK) as a molecular marker. The next eight lanes contain representative *Camk2b* bis-PCR products, which amplified at the predicted amplicon size of 210bp. The final lane contains the negative control.

3. DAB1 Western Supplement

A. Fractional Validation

We first confirmed that the immunoreactive species detected by the selected anti-DAB1 antibody (Rabbit monoclonal, ab68461, Abcam, Cambridge, UK) was a single band of the predicted molecular weight, in the anticipated cellular fraction (Supplementary Figure S2A). For this, cytosolic (C) and membrane (M) fractions were used at the same protein loading (50μg) per well. There was a clear enrichment for the anti-DAB1 band in the cytosolic fraction as expected given that DAB1 is primarily cytosolic (Trotter et al., 2013). This anti-DAB1 band was found to migrate at a molecular weight of ~80kDa (Supplementary Figure S2A) supporting the literature which has described the primary rat brain DAB1 isoform of 80kDa [88-93].

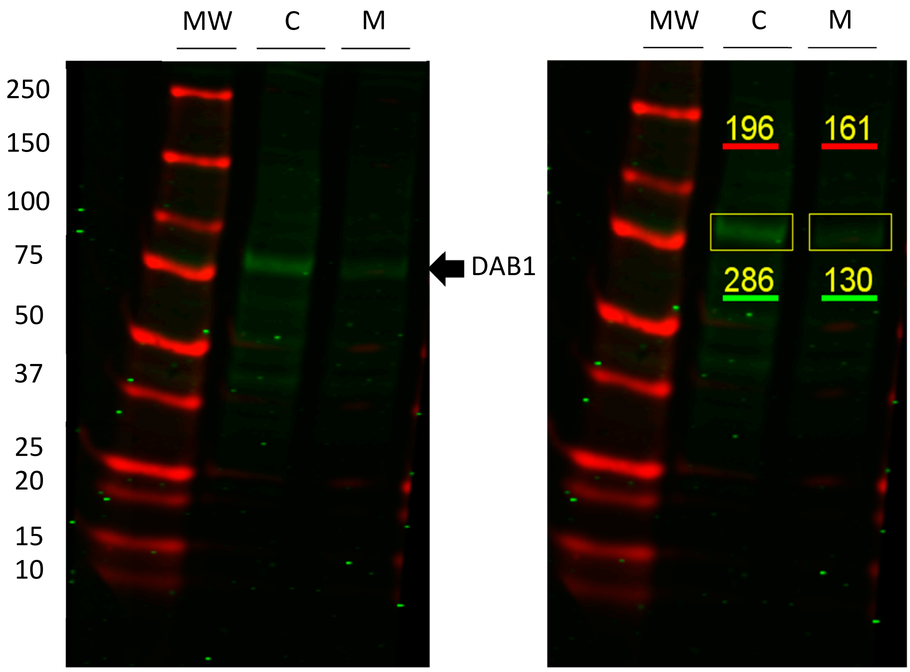

Supplementary Figure S3A. DAB1 fractional validation blot for rabbit monoclonal, anti-DAB1, ab68461, Abcam. Western blots were performed as described in Materials and Methods using precast Mini-PROTEAN Tris-Glycine eXtended (TGX) Gels with 5μL Precision Pus Protein™ All Blue Prestained Protein Standards. Secondary IRDye® antibodies were used for infrared detection. Imaged above is the DAB1 signal, showing higher expression in the cytosolic fraction (left). Abbreviations: MW, molecular weight ladder; C, cytosolic fraction; M, membrane fraction. Molecular weight (kDa) is indicated on the left.

B. Positive and Negative controls

Following the initial validation, we aimed to include a positive and negative control (excluding the primary antibody) assessment. For our positive control, the same developmental samples were evaluated as those used in our negative control gel. DAB1 was expected to be more highly expressed in fetal and early development compared to adulthood [28], acting as a further validation of the antibody specificity (Supplementary Figure S2B). A positive western blot using samples across the developmental timeline showed a clear enrichment of DAB1 prenatally, bolstering confidence in antibody specificity, while the negative control, treated under the same conditions but without the addition of the primary anti-DAB1 antibody, showed abolition of signal supporting the specificity of this immunoreactive band.

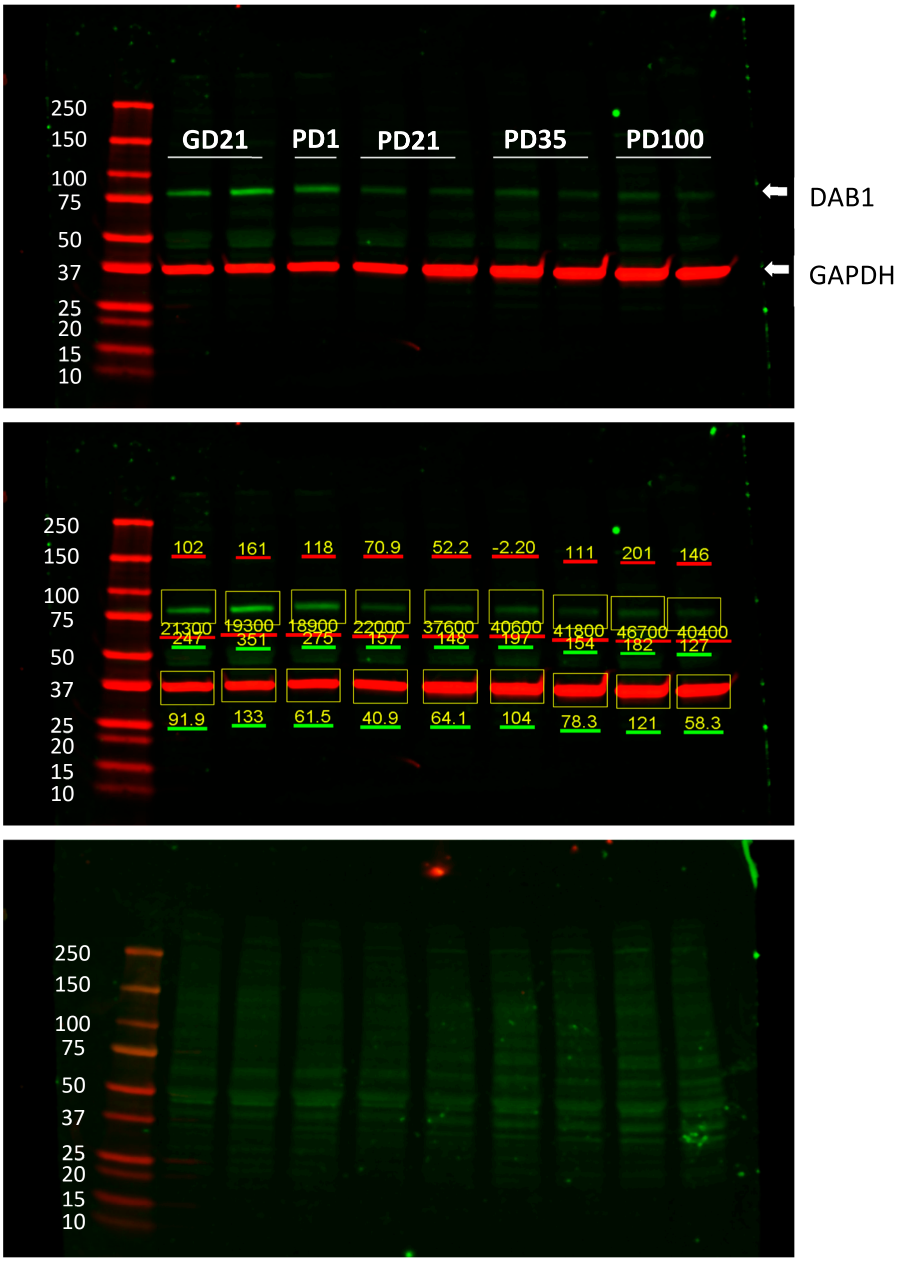

Supplementary Figure S3B. DAB1 positive and negative validation blots for rabbit monoclonal, anti-DAB1, ab68461, Abcam. Western blots were performed as described in Materials and Methods using precast Mini-PROTEAN Tris-Glycine eXtended (TGX) Gels with 5µL Precision Pus Protein™ All Blue Prestained Protein Standards for molecular weight analysis. Secondary IRDye® antibodies were used for infrared detection. Imaged above is DAB1 signal (top) showing greater expression early in development, with abolition of signal in absence of primary antibody (bottom). Samples were loaded at 75µg/well to observe relative expression across the developmental timepoints. Molecular weights (kDa) are indicated on the left.

C. Optimisation

To identify the linear range of the assay [94], we tested protein loading (per well) inputs of 25µg, 50µg, 75µg and 100µg, with antibody dilutions of 1:500, 1:1000 and 1:2000. Plotting densitometry of immunoreactive signal against both protein concentration and antibody dilution to determine the linear range of the assay. This optimisation determined, as expected, different conditions depending on developmental stage, with a protein loading of 50µg (GD21-PD1) and 75µg (PD21-100) birth at 1:1000 antibody dilution achieved the linear phase of the assay.

D. Phospho-DAB1 trial

Following quantification of total DAB1, we aimed to identify an antibody to quantify phospho-DAB1. We selected phospho-antibodies from three different suppliers and trialled them on cytosolic lysate samples (PD1-100; Supplementary Figure S2D). No band of the correct size could be identified across any of the samples or conditions we used (Table S2D).

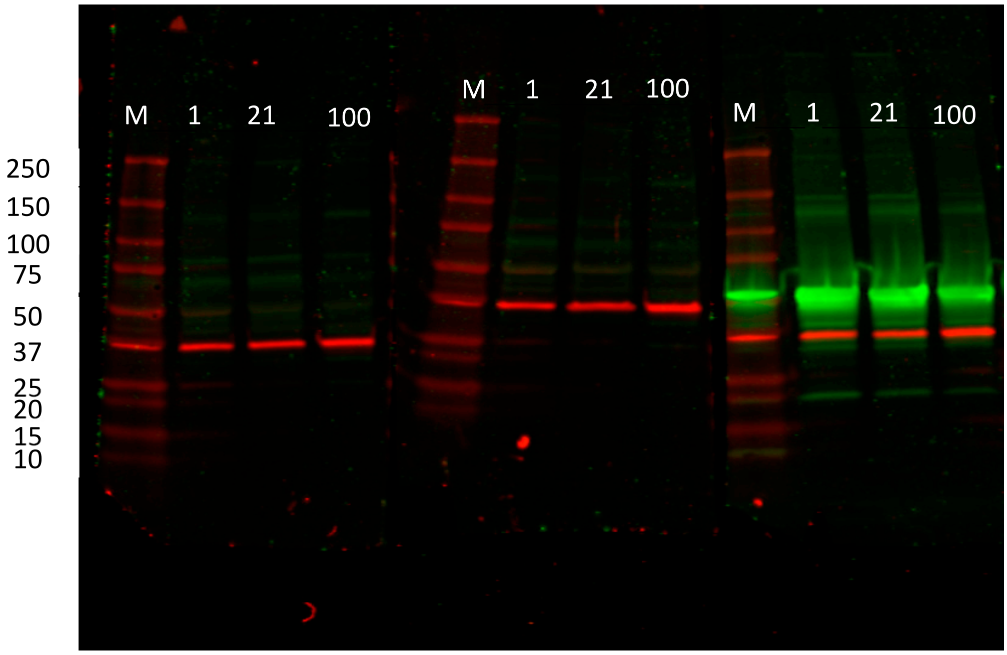

Supplementary Figure S3D. phospho-DAB1 trials. Western blots were performed as described in Materials and Methods using precast Mini-PROTEAN Tris-Glycine eXtended (TGX) Gels with 5µL Precision Pus Protein™ All Blue Prestained Protein Standards for molecular weight analysis. Secondary IRDye® antibodies were used for infrared detection. Samples were loaded at 100µg/well for each of the developmental timepoints (with PD indicated above lane). Image above shows a lack of immunoreactive signal at the predicted size with any of the three antibodies. Molecular weights (kDa) are indicated on the left. Antibodies (Left to right: Cell Signalling Technology, 3327S (left; 1:500); St John's Laboratory, STJ196282 (middle; 1:500); Abcam, ab78200 (right; 1:500)).

Supplementary Table S3D. phospho-DAB1 trials

Methodological stage	Conditions trialled
Primary antibody dilution	1:200; 1:500, 1:1000
Primary antibody incubation	Overnight: 4°C; room temperature<br>3 h: 4°C; room temperature
Sample dilutions	50, 75, 100, 200µg/well
Blocking buffer	2% and 5% BLOTTO<br>5% BSA in 1XPBS.

4. Object Location Test

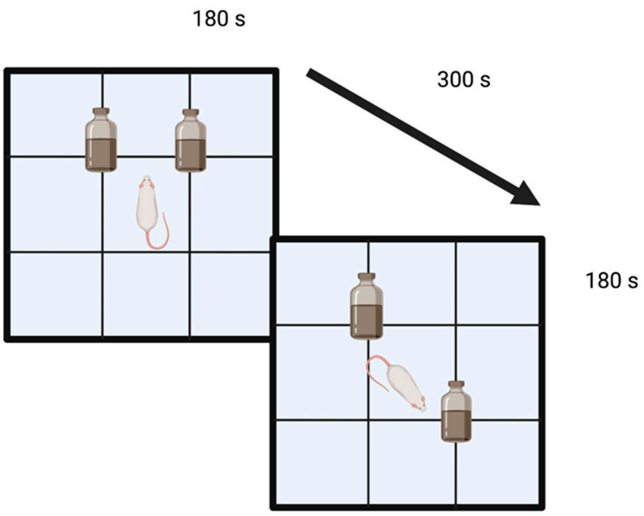

Supplementary Figure S4. Object Location Task. The figure above illustrates the acquisition (top) and retention phase (bottom) of the object location task. After 180 s of free exploration of the two identical items, animals were removed and placed into home cages. After 300 s, they were returned, and offspring were allowed to explore copies of the identical items with the location of one having been altered. Image was created with BioRender.com

5. Object-In-Place Test

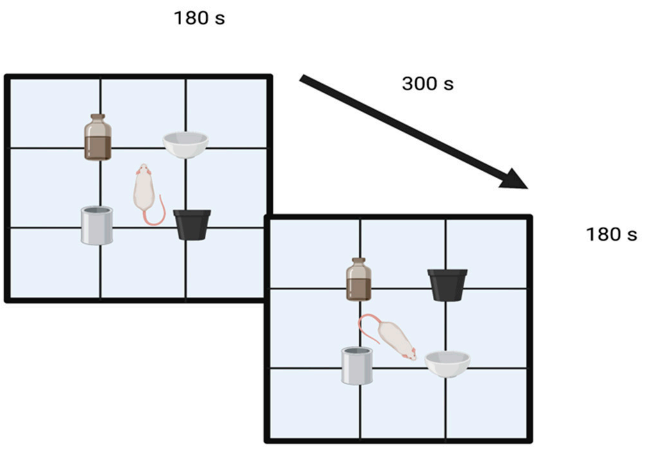

Supplementary Figure S5. Object-In-Place Task. The figure illustrates the acquisition (top) and retention phase (bottom) of the Object-in-Place task. After 180 s of free exploration of four items, animals were removed and placed into home cages. After 300 s, individuals were returned with the location of two having been swapped. Image was created with BioRender.com
